# Role and mechanism of the Th17/Treg cell balance in the development and progression of insulin resistance

**DOI:** 10.1007/s11010-019-03561-4

**Published:** 2019-06-19

**Authors:** Limin Tao, Huanbing Liu, Yanfeng Gong

**Affiliations:** 1 Department of Obstetrics, The First Affiliated Hospital of Nanchang University, No. 17, Yongwai Zheng Street, Nanchang, Jiangxi China; 2 Department of Geriatrics, The First Affiliated Hospital of Nanchang University, No. 17, Yongwai Zheng Street, Nanchang, Jiangxi China

**Keywords:** Th17 cells, Treg cells, IL-6, Insulin resistance

## Abstract

The pathogenic mechanism of insulin resistance and associated diseases such as metabolic syndrome and diabetes remains unclear. Since inflammatory cytokines secreted by T cells play an important role in immune system homeostasis, we evaluated the role of interleukin-6 (IL-6) and the Th17/Treg balance in insulin sensitivity and the underlying mechanism in a rat model. After establishing an insulin-resistant rat model, the rats were injected with anti-mouse IL-6R receptor antibody (MR16-1) to block IL-6. Adipose tissue and blood samples were obtained for the analysis of cytokines, Th17 and Treg markers, and insulin sensitivity blood parameters, for comparisons with those of the normal control group, IL-6-blocked control group, and insulin resistance control group. In the insulin resistance control group, the expression levels of IL-6, RORγt, and IL-17 increased, whereas those of IL-10, FoxP3, and CD4+CD25+Treg decreased. Insulin sensitivity decreased, whereas glucose, total serum cholesterol, triglycerides, and free fatty acid levels significantly increased. However, the completely opposite effects for all parameters were detected in the insulin resistance IL-6-blocked group. Insulin resistance can cause inflammation and an imbalance in Th17 cells/Treg cells. IL-6 can restore this imbalance and play an important role in the development and progression of insulin resistance.

## Introduction

Insulin resistance (IR) refers to a pathophysiologic condition in which there is a decrease in insulin-mediated glucose uptake and utilization in insulin-sensitive tissues and cells such as the liver, skeletal muscle, and fat tissues and cells. Consequently, there are varying degrees of decreased insulin sensitivity in these cells and tissues. In recent years, with improved standards of living, the incidences of IR along with its associated diseases such as metabolic syndrome and diabetes have increased significantly. Moreover, there is an increasing prevalence of IR and related diseases in younger populations. However, the current treatment effect is not satisfactory, and the underlying pathogenesis is not fully understood.

Recent studies have shown that a cytokine imbalance plays an important role in the pathophysiological process of IR and its related diseases. When an inflammatory response is initiated, the body secretes many inflammatory factors such as tumor necrosis factor-alpha (TNF-α), interleukin (IL)-6, and C-reactive protein (CRP) to protect itself [1]. Indeed, the plasma levels of these inflammatory factors are significantly elevated in people with metabolic syndrome. For example, in patients with hypertension, hyperlipidemia, atherosclerosis, type 2 diabetes, and obesity, plasma levels of TNF-α and IL-6 are significantly elevated [2]. TNF-α expression is also significantly increased in the plasma of obese model animals [3]. These observations suggest that inflammatory factors are involved in IR.

Various T cell subpopulations regulate the cytokine balance of the body and secrete inflammatory factors, suggesting a link between T cell regulation in the pathogenesis of IR and its related diseases. T helper cell 17 (Th17) is a newly discovered type of T helper cell [4], which has been shown to be involved in the development and progression of inflammation and many autoimmune diseases through the secretion of IL-17A/F, IL-22, IL-21, TNF-α, and other inflammation-related cytokines [5]. Th17 cell differentiation is largely regulated by the transcription factor RORγt [6]. Regulatory T cells (Tregs) are another unique T lymphocyte subset in the body that secretes IL-4, IL-10, and TGF-β, which is regulated by the factor foxhead box P3 (FoxP3). Tregs inhibit effector T lymphocytes, play a role in a variety of immune responses, and are closely related to the pathogenesis or immune status of a variety of immune diseases [7].

Among these inflammatory cytokines elevated in IR, IL-6 has been reported to play a key role in promoting Th17 cell differentiation via its synergistic induction with TGF-β [8]. The presence of TGF-β alone is not sufficient to induce the differentiation of primary T cells into Th17 cells but can induce their differentiation into Tregs [9]. Large numbers of Th17 cells are only formed when both IL-6 and TGF-β are present, and these cytokines can thus work synergistically [10]. Hanidziar and Koulmanda [11] suggested that the functions of Th17 cells and Tregs are actually antagonistic to each other: Th17 cells appear to be the promoters of immune diseases, whereas Tregs can alleviate the symptoms of immune diseases induced by Th17 cells. This antagonism results in a relatively stable innate immune system that requires coordination for their differentiation, which mainly depends on the state of the innate immune system and the production of IL-6. When the body is in a stable state or when there is no inflammatory damage, TGF-β produced by the immune system inhibits the proliferation of effector T cells and induces FoxP3+ Tregs, thereby maintaining the immune tolerance mechanism. However, in a state of inflammation, the Toll-like receptor-activated immune system will produce a large amount of IL-6, which will inhibit the TGF-β-induced proliferation of Tregs and induce an inflammatory T lymphocyte reaction involving Th17 cells, to ultimately mediate a proinflammatory response. Therefore, Zhou et al. suggested that blocking key cytokines such as IL-6 in vivo might help to transform Th17 cells into Tregs and thus inhibit the development of immune diseases [12].

To test this hypothesis, we evaluated the roles of Th17 cells/Treg cells in IR and the feasibility of blocking IL-6 as an intervention. Toward this end, we suppressed IL-6 production in a rat model of IR using an IL-6 antibody and evaluated the effects on insulin sensitivity, Treg numbers, cytokine and transcription factor levels, and metabolic parameters. These results can provide a novel perspective for understanding insulin sensitivity based on the balance of Th17 cells and Tregs. In turn, this new perspective could offer a theoretical basis for the treatment of IR and its associated diseases.

## Materials and methods

### Grouping and treatment of animals

Thirty-six male clean-grade Wistar rats (6 weeks old, 100–120 g) of inbred lines were obtained from Vital River Experimental Animal Technology Co., Ltd. (Beijing, China) and randomly divided into four groups as follows: the normal control group, IL-6-blocked control group, IR control group, and IR IL-6-blocked group. IR was induced by feeding the rats a sterilized high-fat diet rich in unsaturated fatty acids (fat providing 59% of the calories) for 28 days, and then insulin sensitivity was quantified by the euglycemic hyperinsulinemic clamp technique. To block IL-6, 8 mg of anti-mouse IL-6 receptor antibody (MR16-1; Chugai Pharmaceutical Co., Ltd., Japan) was intraperitoneally injected into each rat on days 0 and 14 after IR model establishment. The same dose of mouse IgG1 (eBioscience, Inc., San Diego, CA, USA) was administered to the control group. After 4 weeks, the rats were killed, and samples of adipose tissue and blood were obtained for testing. These experimental protocols were approved by the Ethics Committee of the First Affiliated Hospital of Nanchang University.

### Expression of RORγt, IL-17, and FoxP3 in adipose tissue

The adipose tissue was lysed in lysis buffer solution. Subsequently, the lysate was immunologically tested after SDS polyacrylamide gel electrophoresis (SDS-PAGE). After transfer to a membrane, the samples were blocked with 1:1000-diluted goat RORγt (S-14) IgG monoclonal antibody, goat IL-17 (G-4) IgG monoclonal antibody, and rabbit FoxP3 IgG monoclonal antibody (all from Santa Cruz Biotechnology, USA), and incubated for 2 h at room temperature. The membranes were then incubated with anti-rabbit, anti-goat, and anti-goat secondary antibody for 1 h at room temperature. The optical density of the band was determined from the X-ray film using the ImageQuant 5.0 software. FoxP3, RORγt, and IL-17 protein expression was standardized against the value of the β-actin band in the corresponding group.

### Insulin sensitivity test

Insulin sensitivity was detected using euglycemic hyperinsulinemic clamps. After anesthesia, catheters were implanted in the left jugular vein. Insulin was started and continued using a precision infusion pump at a rate of 20 mU/kg/min to lower plasma glucose levels to within the physiological range (approximately 5 mmol/L). Physiological blood glucose concentrations were maintained by adjusting the infusion of a 20% glucose solution. Steady state was ascertained when glucose measurements were constant for 20 min at a fixed glucose infusion rate and was achieved within 120–240 min. Steady state was maintained for 45 min and blood samples (10 μL) were taken at 0 and 5 min and then at 10-min intervals after reaching steady state.

### Blood parameters

Blood samples were obtained and stored at 4 °C. Serum was acquired by the centrifugation of blood samples at 3000×*g* for 15 min, immediately after sampling. Peripheral blood glucose, serum total cholesterol, and triglyceride content were measured with the hexokinase method using a Beckman X20 automatic biochemical analyzer. Serum free fatty acids were quantified by HPLC–MS.

### Peripheral blood Treg quantification

A fresh blood sample (100 μL) was obtained from the rats and mixed with 0.25 μg of CD4-FITC and 1.0 μg of anti-mouse CD25-PE in each test tube containing 10^6^ cells. Samples were incubated for 30 min in the dark and then incubated for 20 min further after the addition of red blood cell lysis buffer. The cells were washed with buffer, centrifuged, fixed with a fixation and permeabilization agent, and then incubated for 60 min in the dark. After another wash, the supernatant was decanted, serum was added, the test tubes were sealed, and the samples were incubated for 15 min in the dark. After the addition of 5 μL of FoxP3 antibody, the samples were incubated in the dark for 30 min. The samples were washed twice with permeabilization buffer and centrifuged to discard the supernatant. The cells were resuspended in 300 μL of staining buffer, and the proportion of CD4+CD25+FoxP3+Tregs was measured by flow cytometry.

### Determination of cytokines

Serum levels of IL-6 and IL-10 were measured using enzyme-linked immunosorbent assay kits (eBioscience), as per the manufacturer’s instructions.

### Statistical analysis

Data analysis was performed using SPSS 17.0 software. Measurement data are expressed as the mean ± standard deviation and were compared among groups with an independent-sample *t* test. Correlation analysis was performed based on the Spearman’s correlation. *P* < 0.05 was considered statistically significant.

## Results

### Expression of RORγt, IL-17, and FoxP3 in adipose tissue

**Fig. 1:**
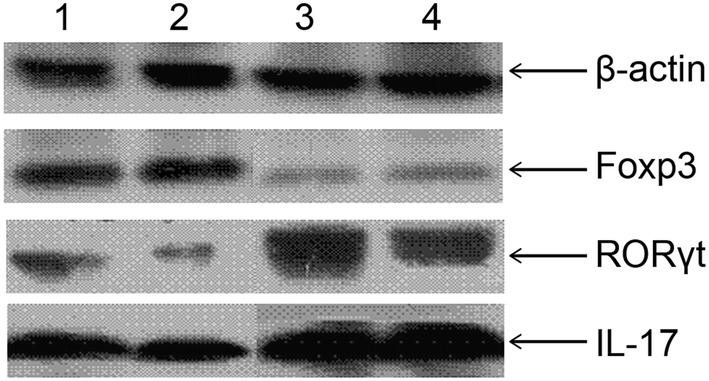
Expression of RORγt, IL-17, and FoxP3 protein in the adipose tissue of rats by Western blot. Lane 1, control group; 2, IL-6-blocked control group; 3, insulin resistance control group; 4, insulin resistance IL-6-blocked group

The expression levels of RORγt, IL-17, and FoxP3 proteins in adipose tissue significantly differed among the groups (*P* < 0.001). Further comparison demonstrated no significant differences between the control group and IL-6-blocked group. The expression of FoxP3 in the IR plus IL-6-blocked group was significantly higher than that in the groups without IR (*P* < 0.05). However, the expression levels of RORγt and IL-17 were lower in the IR plus IL-6-blocked group than in the groups without IR (*P* < 0.05) (Table 1; Fig. 1).

**Table 1:** Expression of RORγt, IL-17, and FoxP3 in adipose tissue of rats

Group	*N*	FoxP3	RORγt	IL-17
Normal control	10	0.56 ± 0.04	0.52 ± 0.12	0.43 ± 0.07
IL-6-blocked control	10	0.68 ± 0.11	0.4 ± 0.02	0.36 ± 0.01
Insulin resistance control	10	0.14 ± 0.23^a^	0.93 ± 0.24^a^	0.98 ± 0.04^a^
Insulin resistance plus IL-6-blocked	10	0.39 ± 0.15^a,b^	0.72 ± 0.08^a,b^	0.66 ± 0.10^a,b^

^a^*P* < 0.01 versus normal control and IL-6-blocked control groups

^b^*P* < 0.05 versus insulin resistance control group

### Insulin sensitivity and peripheral blood glucose, insulin, total cholesterol, triglycerides, and free fatty acid levels

There were significant differences in insulin sensitivity and blood glucose, insulin, total cholesterol, triglyceride, and free fatty acid levels among the groups (*P* < 0.001; Table 2).

**Table 2:** Determination of insulin sensitivity among rat treatment groups

Group	*N*	Insulin sensitivity index (ISI)	GIR_60–120_ [mg/(kg/min)]
Normal control	10	− 3.75 ± 0.17	7.12 ± 0.56
IL-6-blocked control	10	− 3.75 ± 0.17	7.12 ± 0.56
Insulin resistance control	10	− 5.47 ± 0.23^a^	12.11 ± 0.48^a^
Insulin resistance plus IL-6-blocked	10	− 4.26 ± 0.19^a,b^	15.01 ± 1.52^a,b^

^a^*P* < 0.01 versus normal control and IL-6-blocked control groups

^b^*P* < 0.05 versus insulin resistance control group

Further comparison showed no significant differences in insulin sensitivity, peripheral blood glucose, total cholesterol, triglyceride, and free fatty acids between the non-IL-6-blocked and IL-6-blocked control groups. However, both of the IR groups (non-IL-6-blocked and IL-6-blocked) had significantly higher insulin sensitivity and peripheral blood glucose, insulin, total cholesterol, triglyceride, and free fatty acid levels than the control groups. Moreover, the IR IL-6-blocked group had significantly higher insulin sensitivity than all other groups. The levels of blood glucose, insulin, total cholesterol, triglycerides, and free fatty acids 2 h postprandial in the IR IL-6-blocked group were significantly lower than those in the non-IL-6-blocked IR group (Table 3).

**Table 3:** Peripheral blood glucose, insulin, total cholesterol, triglycerides, and free fatty acids levels in rat treatment groups

Group	*N*	Blood glucose (mmol/L)		Insulin (μU/mL)	Total cholesterol (mg/dL)	Triglycerides (mg/dL)	Free fatty acids (μmol/L)
		Fasting	2 h post-prandial				
Normal control	10	4.87 ± 0.52	5.76 ± 0.70	20.34 ± 4.54	0.83 ± 0.07	0.58 ± 0.21	0.98 ± 0.11
IL-6-blocked control	10	4.87 ± 0.52	5.76 ± 0.70	20.34 ± 4.54	0.83 ± 0.07	0.58 ± 0.21	0.98 ± 0.11
Insulin resistance control	10	5.39 ± 0.67^a^	10.53 ± 0.59^a^	31.87 ± 9.46^a^	1.52 ± 0.11^a^	1.72 ± 0.43^a^	2.83 ± 0.32^a^
Insulin resistance plus IL-6-blocked	10	5.20 ± 0.48^a,b^	8.70 ± 1.27^a,b^	25.42 ± 6.35^a,b^	1.01 ± 0.13^a,b^	1.04 ± 0.20^a,b^	1.42 ± 0.21^a,b^

^a^*P* < 0.01 versus normal control and IL-6-blocked control groups

^b^*P* < 0.05 versus insulin resistance control group

### Peripheral blood CD4+CD25+Tregs and cytokines

There were significant differences in the expression of CD4+CD25+Tregs and the cytokines IL-6 and IL-10 in the peripheral blood among groups (*P* < 0.001). Further comparison showed no differences between the normal control and IL-6-blocked control groups. However, the IR IL-6-blocked group had significantly higher levels of CD4+CD25+Tregs and IL-10 and significantly lower IL-6 levels than the non-IL-6-blocked IR control group (Table 4).

**Table 4:** Proportion of CD4+CD25+Tregs and expression of IL-6, and IL-10 in peripheral blood of rat treatment groups

Group	*N*	CD4+CD25+Tregs (%)	IL-6 (ng/L)	IL-10 (ng/L)
Normal control	10	4.11 ± 1.03	28.38 ± 2.16	255.43 ± 18.69
IL-6-blocked control	10	5.63 ± 0.78	23.42 ± 2.08	255.43 ± 18.69
Insulin resistance control	10	1.39 ± 0.43^a^	78.4 ± 11.54^a^	125.94 ± 1.78^a^
Insulin resistance plus IL-6-blocked	10	2.95 ± 0.89^a,b^	45.89 ± 6.38^a,b^	196.10 ± 9.37^a,b^

^a^*P* < 0.01 versus normal control and IL-6-blocked control groups

^b^*P* < 0.05 versus insulin resistance control group

## Discussion

Many studies have shown that proinflammatory cytokines including TNF-α, IL-6, IL-1β, CRP, and NF-κB can lead to IR and the development of related diseases such as metabolic syndrome and diabetes [13], either alone or in combination, via various pathways [14]. The inflammatory response activates macrophages, which then activates the inflammatory factors. In particular, CRP, TNF-α, and IL-6 have synergistic effects and individual effects to activate NF-κB, thereby promoting nitric oxide synthesis to enhance the inflammatory response. TNF-α also induces MCP-1 production, which activates and induces the chemotactic migration of macrophages [15]. This positive feedback loop ultimately results in the inhibition of insulin signaling and insulin sensitivity. Inflammatory factors also increase the levels of peripheral free fatty acids through lipolysis, which further aggravates IR. This forms a perpetual cycle, in which uncontrolled levels of blood sugar and blood lipids can lead to many complications. Our study confirmed that during IR, IL-10 expression is decreased and IL-6 expression is increased. Moreover, Th17 cell markers such as RORγt and IL-17 were increased, and Treg markers such as FoxP3 and CD4+CD25+Tregs were decreased. Insulin sensitivity was reduced, whereas blood sugar, total serum cholesterol, triglycerides, and free fatty acids levels were also significantly increased in IR states. However, the inhibition of IL-6 resulted in increased IL-10 expression and decreased IL-6 expression, reduced levels of Th17 cell markers, and an increase in Treg markers. Moreover, insulin sensitivity appeared to increase, whereas blood glucose, serum total cholesterol, triglycerides, and free fatty acids level were significantly reduced. Overall, these results indicate that IR can induce an inflammatory response, which is closely related to changes in blood sugar and blood lipids. Therefore, IR can be improved when this inflammatory response is blocked.

The effects of Th17 cells and Tregs are known to be functionally antagonistic. Thus, the occurrence of immune diseases is considered largely due to the decrease in the number or function of Tregs, whereas Th17 cell function continues to increase resulting in damage to the body through attacks on self-tissues [16]. Many studies have found that an imbalance in the number and function of Th17 cells/Tregs might be involved in a variety of immune system diseases such as systemic lupus erythematosus [17], allergic diseases [18], and encephalomyelitis [19]. Consistently, our results demonstrated an imbalance of Th17/Tregs in an inflammatory IR state. Since IL-6 plays an important role in the differentiation of Th17 and Treg cells and can restore their imbalance, IL-6 also plays a crucial role in the development of IR. Thus, IL-6 could be a good target for the development of a novel intervention for IR and related diseases.

## Data Availability

The datasets generated during or analyzed during the current study are available from the corresponding author on reasonable request.
